# Effectiveness of the Use of Virtual Reality Rehabilitation in Children with Dyslexia: Follow-Up after One Year

**DOI:** 10.3390/brainsci14070655

**Published:** 2024-06-27

**Authors:** Giuseppa Maresca, Francesco Corallo, Maria Cristina De Cola, Caterina Formica, Silvia Giliberto, Giuseppe Rao, Maria Felicita Crupi, Angelo Quartarone, Alessandra Pidalà

**Affiliations:** IRCCS Centro Neurolesi Bonino Pulejo, S.S 113, C. da Casazza, 98124 Messina, Italy; giusy.maresca@irccsme.it (G.M.); francesco.corallo@irccsme.it (F.C.); katia.formica@irccsme.it (C.F.); silvia.giliberto@irccsme.it (S.G.); giuseppe.rao@irccsme.it (G.R.); maria.crupi@irccsme.it (M.F.C.); angelo.quartarone@irccsme.it (A.Q.); alessandra.pidalà@irccsme.it (A.P.)

**Keywords:** dyslexia, virtual reality, learning disabilities

## Abstract

Dyslexia is a common learning disorder that hinders reading fluency and comprehension. Traditional treatments can be tedious for children, limiting their effectiveness. This study investigated the one-year effects of rehabilitation treatment with a virtual reality rehabilitation system (VRRS) on children with dyslexia. Twenty-four children were divided into control (CG) and experimental (EG) groups. The CG underwent conventional neuropsychological treatment (CNT), while the EG underwent VR neurorehabilitation training (VRNT) using the VRRS. Neuropsychological evaluation was conducted before treatment, after six months, and again after one year for both groups. Compared to the control group, children who received VR training showed significant improvement in reading skills, especially in non-word reading and reading speed, even after one year without further VR intervention. VRRS can improve treatment adherence and minimize symptoms by offering engaging activities for children. These findings suggest VRRS may be a valuable tool for dyslexia rehabilitation with long-lasting effects.

## 1. Introduction

Dyslexia is one of the specific learning disorders that manifest as a difficulty in learning to read, write and calculate. In particular, dyslexia is a neurodevelopmental disorder that affects reading skills [1]. Although dyslexia is fairly common, its prevalence is uncertain, ranging from less than 5 percent to 20 percent [2,3,4], due to several factors that make it difficult to estimate, such as environmental variables (e.g., regions, socioeconomic status) and other factors (e.g., grade, sub-deficit) [3,4]. The causes of dyslexia are not fully understood to date, although scientific evidence is in favor of a genetic and neurobiological basis that combines with environmental risk factors [5,6,7,8]. In the absence of an ascertainment of the disease and without knowing the reasons for the difficulties in the school environment, the dyslexic child may feel inadequate and show problems of self-esteem [9]. Treatment of dyslexia involves early management shared between the family, the child and the caregivers involved [10,11]. In general, rehabilitation care should be started as soon as possible by specialized operators during the attendance of elementary and middle schools. For dyslexia, specialized interventions aimed to improve the speed and correctness of reading are recommended, as well as those aimed to automate the conversion processes between written and oral language, in addition to activities aimed to promote phonological awareness and the learning of the conversion rules between graphemes (letters) and phonemes (sounds) [12]. Although there are different types of rehabilitation interventions to reduce the symptoms of dyslexia in children [13], it is necessary to consider the possible subject’s comorbidities when choosing the most suitable intervention [14].

The use of new technologies in rehabilitation for dyslexia is constantly increasing [15]. In recent years, in fact, high-tech technologies have been used in studies concerning learning disabilities for enhancement in writing and reading skills [16,17,18]. These technologies can support the learning process by providing immediate feedback and an individualized learning environment [17,18,19], providing greater adherence to treatment [20]. Other research has shown that intensive and short PC-based or Wii station treatments can improve reading skills in dyslexic children, as well as visual attention skills, spatial cognition, auditory spatial attention and reactivity [21,22,23,24].

In recent years, the virtual reality rehabilitation system (VRRS) has been developed, which aims to improve cognitive and linguistic deficits with specific modules in patients with neurological disorders [25,26,27,28,29]. The use of virtual reality, according to some studies [30,31,32], could be an effective rehabilitation option to improve the cognitive profiles of children with reading difficulties. In fact, the use of virtual reality can be a promising approach to address multiple cognitive and linguistic aspects underlying normal and impaired reading [19,30,31]. Our previous study, conducted on patients with dyslexia [20], showed an improvement in cognitive domains and some linguistic domains, i.e., the scores of words in reading tests and homophonic writing, after a rehabilitation training of about 6 months, and the results were significantly higher in the experimental sample subjected to rehabilitation treatment with VRRS compared to the control sample subjected to traditional neuropsychological treatment. The study showed that intervention with VRRS led to better outcomes through the use of virtual reality probably due to the facilitation of active exploration and engagement in the motivation and fun that VRRS brings. There are no ongoing studies evaluating the efficacy over time of rehabilitation treatment with VRRS in patients with dyslexia. Aiming to fill this gap, in this study, we evaluated the one-year effects of rehabilitation treatment with VRRS in children with dyslexia.

## 2. Materials and Methods

A total of 28 children (15 females and 13 males) diagnosed with dyslexia were enrolled at the Child Neuropsychiatry Unit of the IRCCS “Bonino Pulejo” Neurolesi Center in Messina. Inclusion criteria were as follows. (1) A diagnosis of dyslexia according to the four criteria of the Diagnostic Statistical Manual, Fifth Edition (DSM-5): (a) presence of at least six symptoms of learning difficulty for at least 6 months despite the provision of extracurricular help or targeted instruction; (b) difficulties in literacy and mathematical skills such as reading a single word, understanding reading, writing and spelling, calculation and mathematical reasoning; (c) deficits in fundamental academic skills with poor academic achievement and lagging behind in terms of age and intellectual ability compared to the peer group; and (d) the delay in academic achievement is not due to another medical condition, intellectual disability, or inappropriate academic instruction (American Psychiatric Association 2013; Diagnostic and Statistical Manual of Mental Disorders, 5th ed. Washington). (2) The absence of serious medical and psychiatric conditions. Patients were randomized into either the control (CG: *n* = 14) or the experimental (EG: *n* =14) group. However, 4 of them were excluded because of missing data due to dropout in the last phase of the study. Therefore, 24 patients (12 females and 12 males), aged 8–14 years, were included in this longitudinal study. The CG underwent conventional neuropsychological treatment (CNT), while the E underwent VR neurorehabilitation training using VRRS (VRNT). All subjects underwent a neuropsychological evaluation at the beginning (T0) and at the end of the six-month rehabilitation program (T1), which consisted of 72 sessions of 60 min each, three times a week. Follow-up (T2) was performed after 12 months to investigate the long-term maintenance of the results obtained. The local ethics committee approved the study (protocol code 15/2019 and date of approval 25 September 2019), and informed consent was obtained from all subjects involved in the study and their parents.

### 2.1. Neuropsychological Assessment

All subjects were assessed with the Wechsler Intelligence Scale for Children-IV (WISC-IV) [33] and the Italian Battery for the Evaluation of Dyslexia and Dysorthography (DDE) [34] to evaluate the level of competence acquired in reading and writing and the effectiveness of the treatment. WISC-IV evaluates the intellectual abilities of subjects aged between 6 and 16 years and 11 months. The WISC-IV evaluates four cognitive areas, using specific indices: verbal comprehension index (VCI), visual–perceptual reasoning index (PRI), working memory index (WMI) and processing speed index (PSI). In addition, the WISC-IV consists of three composite indices: Global Intelligence Quotient (IQ), General Ability Index (GAI) and Cognitive Competence Index (CCI). One of the clinical applications of the WISC-IV is the cognitive assessment of DSA. In fact, to diagnose a DSA, an IQ of no less than 85 and a discrepancy between IQ and scholastic performance are required (Istituto Superiore di Sanità, 2011). The instrument, underlining the importance of WMI and PSI, is fundamental for the diagnosis and study of DSA. Many studies have shown how DSA are associated with deficient performance in these two cognitive functions [35,36]. The DDE battery is part of the standard diagnostic protocol for the evaluation of learning disabilities in reading, writing and arithmetic, approved by the Italian Dyslexia Association, and helps to assess reading and writing proficiency as well as monitor progress to compare diagnostic and therapeutic results. This test is instrumental in examining reading and writing difficulties during a diagnosis of DSA, monitoring the development of reading and writing skills and comparing the results of diagnostic and rehabilitation treatment. The DDE battery includes eight subtests: five evaluate reading skills (grapheme naming, reading words and non-words, understanding sentences with homophones and correcting homophones) and three evaluate writing skills (dictating words and non-words and dictation of sentences with homophones).

### 2.2. Decription of Treatments

All patients underwent the same amount of rehabilitation treatments but using different tools. The CG underwent CNT, administered in individual sessions using a face-to-face interaction between therapist and patient with paper-and-pencil activities, while the EG performed the same activities using a virtual reality rehabilitation system (VRRS, Khymeia, Padua, Italy). The tasks were the same for both groups, but the difficulty and duration varied depending on the needs and objectives to be achieved. Both treatments were aimed at stimulating different cognitive domains (such as memory, attention, language, spatiotemporal orientation, executive functions, calculation and practice).

In this study, we used the cognitive exercise module of the VRRS, which includes more than 50 types of rehabilitation activities [37]. The VRRS cognitive module used in this study consists of a wide range of rehabilitation activities, with more than fifty exercises. Two-dimensional cognitive exercises enable patients to engage with objects and scenarios using a touchscreen or mouse, simulating real-life interaction. The VRRS system is designed to enhance feedback to the CNS (central nervous system) by providing intensive, repetitive and task-oriented exercises within a virtual environment. This approach helps patients to develop an understanding of outcomes and performance quality. As a result, it can trigger “reinforcement learning”, leading to improved performance quality [27,38]. Furthermore, training in a playful virtual reality environment is more stimulating and motivating for patients and consequently promotes its treatment adherence.

### 2.3. Statistical Analysis

Data were analyzed using the R version 4.3.0, at a 95% confidence level, and an alpha = 0.05 was set as level of statistical significance. Given the small sample size and the ordinal nature of DDE variables, a nonparametric approach was adopted. Thus, the Mann–Whitney U test was used to assess differences between groups, whereas the chi-squared test was applied in proportions.

The Friedman test was applied to perform a non-parametric one-way repeated measures to evaluate differences in DDE scores among assessment times (i.e., T0, T1, and T2) for EG and CG each. For a post hoc analysis, we used the Wilcoxon–Nemenyi–McDonald–Thompson test, in which *p*-values were already corrected for multiple testing.

The Levene test was used to assess homoscedasticity, whereas the lme4 package of R was used to perform a linear mixed-effects analysis of the relationship between WISC-IV outcome and treatment. The model included the two levels variable “group” (EG, CG) and the three-level variable “assessment” (T0, T1, T2) as fixed effects, while the subject’s variability as random effect, and also included correlated intercepts and slopes for the fixed factors and interactions between fixed effects. This model was compared with the null model (i.e., without the effect “group”) by means of the likelihood ratio tests to assess its validity.

## 3. Results

No significant differences were found between the two groups in either demographic characteristics (Table 1) or outcome scores (Table 2) at baseline.

As reported in Table 3, overall, the repeated-measures analysis showed smaller significant differences when comparing scores between T0 and T2 in EG patients than in controls, finding significant changes between T0 and T2 for all clinical tests in the EG, as opposed to the CG. In particular, we found significant over-time improvements in the non-word reading (F(2) = 21.26; *p* < 0.001) and speed of reading (F(2) = 17.90; *p* < 0.001) only in EG. No significant differences between T1 and T2 emerged in both EG and CG.

**Figure 1:**
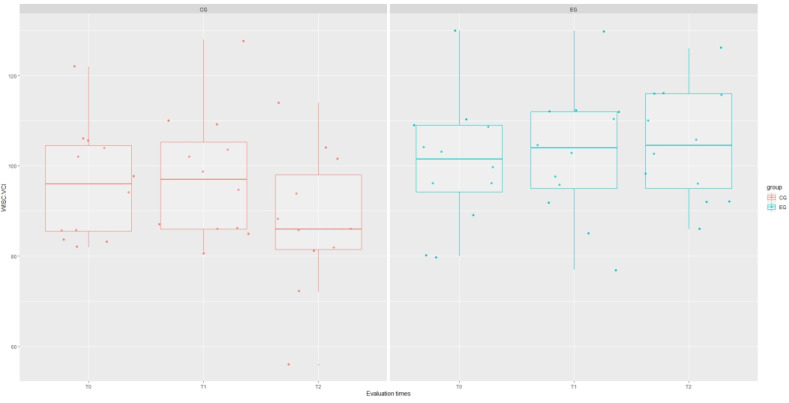
Boxplot of WISC.VCI (verbal comprehension index) scores at each assessment time with grouping with respect to treatment type.

**Figure 2:**
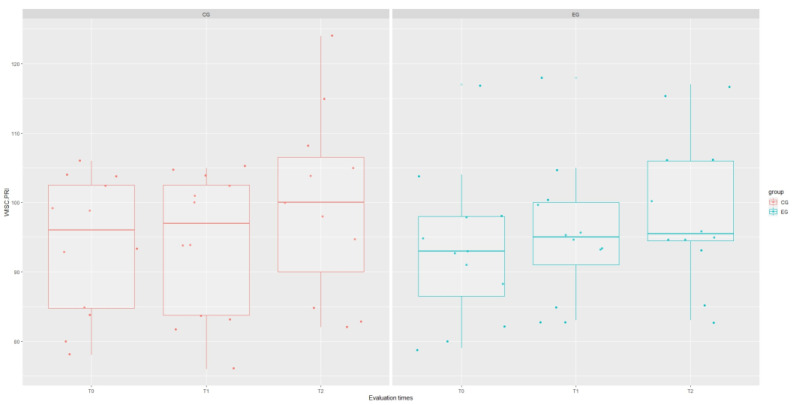
Boxplot of WISC.PRI (visual–perceptual reasoning index) scores at each assessment time with grouping with respect to treatment type.

**Figure 3:**
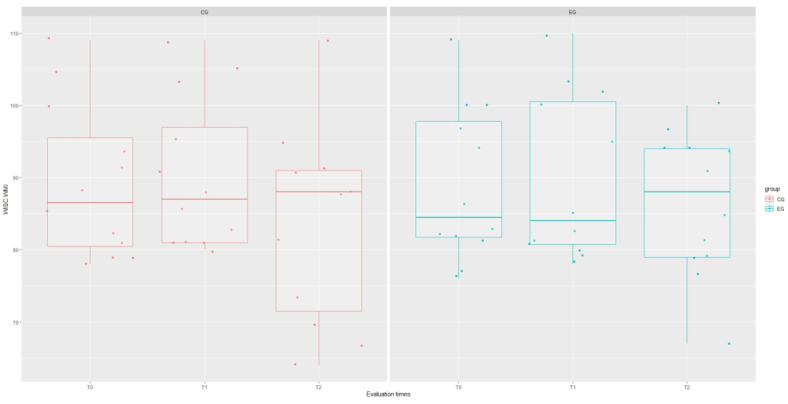
Boxplot of WISC.WMI (working memory index) scores at each assessment time with grouping with respect to treatment type.

**Figure 4:**
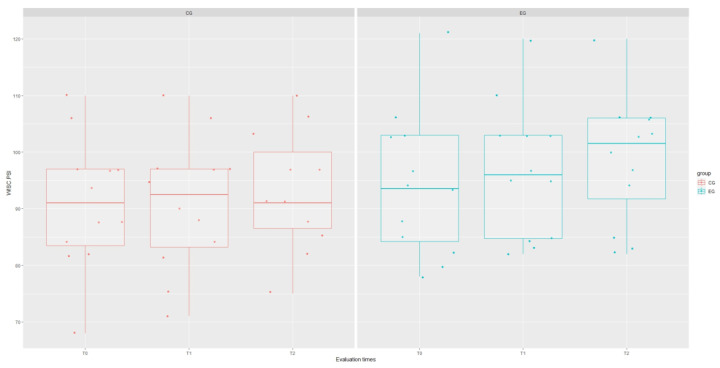
Boxplot of WISC.PSI (processing speed index) scores at each assessment time with grouping with respect to treatment type.

**Figure 5:**
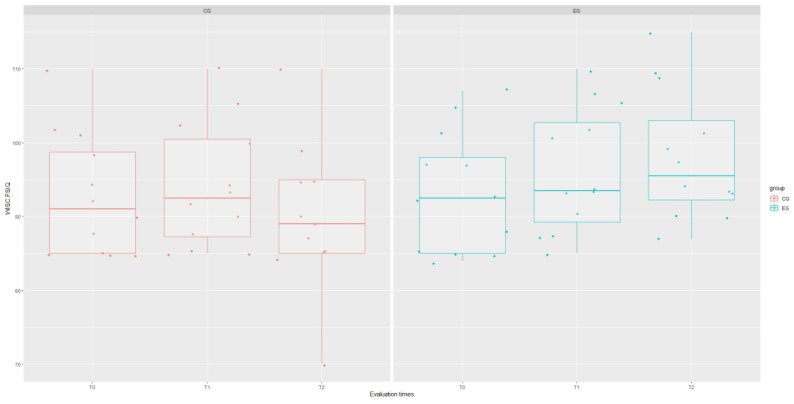
Boxplot of WISC.IQ (Global Intellectual Quotient) scores at each assessment time with grouping with respect to treatment type.

The results of the mixed-effect analysis showed that the type of treatment affected the WISC Verbal Communication Index (VCI) domain (χ^2^(1) = 4.215; *p* = 0.040), increasing the scores of this test in the experimental group by about 8.48 ± 43.78 (*t* = 2.24; *p* = 0.044), as well as the WISC Global Intelligence Quotient (IQ) domain (χ^2^(1) = 8.12; *p* = 0.044), although this significance is influenced by the interaction term “group: time” (*t* = 2.61; *p* = 0.012). However, signs of a stronger recovery trend in EG are evident for each WISC domain, as shown in Figure 1, Figure 2, Figure 3, Figure 4 and Figure 5.

## 4. Discussion

Following our previous study’s findings on the effectiveness of VRRS in improving cognitive function in children with dyslexia [20], this follow-up investigation aimed to assess the long-term effects of VRRS intervention. Traditionally, rehabilitation for dyslexia has relied on paper-and-pencil exercises, which can be tedious and discouraging for children [39]. VRRS offers a more engaging and motivating alternative that can address this challenge. On the whole, our findings showed statistically significant differences after one year in test scores in both groups (i.e., in both subjects who received VRNT and controls who underwent traditional treatment), although the *p*-values of these differences were noticeably smaller in the experimental group. In addition, only EG subjects showed a significant over-time improvement in two key areas of reading: non-word reading and reading speed, as well as in global QI. Improvements in non-word reading within the EG suggest enhanced phonological processing, a fundamental skill for developing fluent reading abilities and overall communication skills. Phonological processing involves the ability to understand the connection between the sounds of a language and the written symbols that represent them [40]. By strengthening this connection, VR training would appear to equip children with dyslexia with better tools to decode unfamiliar words. Additionally, the observed gains in reading speed within the EG point towards increased efficiency in deciphering written words. This improvement in fluency can significantly enhance a child’s overall reading experience. The positive impact of VRRS can be attributed to several factors. Virtual scenarios provide additional sensory feedback that can lead to changes in synaptic plasticity and promote learning [41]. This enhanced neural activity likely contributes to the effectiveness of VRNT in dyslexia. Furthermore, VR environments may hold promise for improving attention skills, a crucial element in dyslexia rehabilitation [39]. By incorporating cues within the virtual environment, VRNT may prime children for phonological awareness and ultimately improve their decoding skills. Studies have also shown that VRRS interventions benefit children with other communication disorders, such as developmental language disorder, increasing motivation, reducing anxiety, and making therapy more enjoyable for children [20,42]. It has been proven that VRRS helps to boost self-confidence and encourages active participation [43,44]. As a result, children are more likely to adhere to treatment, leading to longer durations of speech therapy. Studies have shown that these benefits are essential in enhancing the effectiveness of speech therapy, improving communication skills, and ultimately leading to better outcomes for children with communication disorders [45]. Similar to findings with action video games for reading intervention [39], our results suggest that a more prolonged VRRS protocol may be useful to achieve lasting improvements in reading performance. Encouragingly, our results demonstrated that the children in the EG who received VRNT in the initial study maintained the observed improvements in cognitive domains even without further VRRS intervention during the one-year follow-up period. This suggests that VRRS may have an advantage over other interventions, as it provides more sustained effects compared to interventions with temporary benefits. The persistence of VRRS-induced cognitive benefits in the EG, even without continued intervention, highlights its potential as a long-term solution.

In line with the literature findings, our results indicate that virtual reality could be a viable rehabilitation option for children with reading difficulties, enhancing the cognitive processes involved [32]. The VRRS system engages various linguistic, visual and attentional processes, integrating these elements into complex tasks like reading. Other researchers also suggest that virtual reality holds promise for improving multiple cognitive and linguistic components essential for both typical and impaired reading, aiding in the automation of reading processes.

*“Furthermore, we hypothesize that rehabilitation with virtual reality systems will increase the child’s interest in treatment adherence, return immediate feedback, and emphasize the playful aspect. Children often experience frustration with the required task, and therefore, personalizing an ad hoc program strengthens self-esteem and cognitive performance”.*

[31,39]

### Strengths and limitations

A crucial aspect of this study lies in its contribution to a previously unexplored area. While prior research has documented the effectiveness of VRNT for dyslexia in the short term, there has been a lack of follow-up studies investigating the intervention’s efficacy over extended periods. This follow-up investigation seeks to fill this gap by providing the first set of data on the impact of VRRS after one year. It is important to acknowledge the limitations of the study design. The relatively small sample size of the study, and the wide age range within such a small sample, requires caution when generalizing these findings to a broader population of children with dyslexia. To further our understanding of the long-term efficacy of VRRS, future studies with a longer follow-up period and a significantly larger sample size are needed. Furthermore, a design where the experimental group receives continued VRNT during the follow-up period compared to a control group would provide a clearer picture of the impact of continued VRRS intervention.

## 5. Conclusions

This follow-up study provides promising evidence for the potential of VRRS in dyslexia rehabilitation. The results of this study provide further evidence that VR interventions, by incorporating elements of active engagement and motivation, can effectively address core reading difficulties in this population. The maintenance of cognitive improvements in the EG, even without continued VRRS intervention, is a positive finding. Incorporating VRRS as an additional therapy can be advantageous in minimizing symptoms by providing engaging and interactive activities that motivate children, leading to better adherence to treatment. It could be useful to implement traditional rehabilitation treatments with interventions based on virtual reality, in order to carry out more intensive, specific and early treatments. Based on the observed advantages of VRRS, we recommend its broad implementation in clinical practice.

## Figures and Tables

**Table 1:** Demographic description of the participants who completed the study.

	All	EG	CG	*p*-Value
*Participants*	24	12 (50.0)	12 (50.0)	-
*Male*	12 (50.0)	7 (58.3)	7 (58.3)	1.00
*Age (years)*	10.2 (1.9)	10.1 (1.9)	10.3 (2.1)	0.88
*Education (years)*	5.2 (1.9)	5.0 (1.7)	5.3 (2.1)	0.84

Legend: experimental group (EG); control group (CG). Continuous variables were expressed as mean (standard deviation), whereas categorical variables as frequencies (percentages).

**Table 2:** Clinical description of the participants who completed the study.

Clinical Assessment	EG	CG	*p*-Value
**DDE**			
*Word Reading Test (CORRECTNESS)* *Word Reading Test (SPEED)*	1.5 [1.0–5.5] 2.0 [1.0–3.0]	1.0 [1.0–2.7] 2.0 [1.0–5.2]	0.605 0.952
*No Word Reading Test (CORRECTNESS)*	1.5 [1.0–4.0]	2.5 [1.0–5.7]	0.503
*No Word Reading Test (SPEED)*	3.0 [2.0–6.2]	2.0 [1.7–7.2]	0.882
*Text Reading Test (CORRECTNESS)*	3.0 [1.7–6.2]	7.0 [3.0–8.0]	0.198
*Text Reading Test (SPEED)*	2.5 [2.0–4.2]	4.5 [2.0–6.0]	0.181
*Word Writing Test (CORRECTNESS)*	2.0 [1.0–3.0]	1.0 [1.0–2.7]	0.399
*No Word Writing Test (CORRECTNESS)*	2.5 [1.0–3.7]	2.0 [1.7–4.2]	0.882
*Homophone Word Writing Test (CORRECTNESS)*	2.5 [1.7–6.0]	1.0 [1.0–2.2]	0.195
**WISC-IV**			
*Verbal Comprehension Index*	101.5 [94.2–109.0]	96.0 [85.5–104.5]	0.452
*Visual–Perceptual Reasoning Index*	93.0 [86.5–98.0]	96.0 [84.7–102.5]	0.602
*Working Memory Index*	84.5 [81.7–97.7]	86.5 [80.5–95.5]	0.999
*Processing Speed Index*	93.5 [84.2–103.0]	91.0 [83.5–97.0]	0.816
*Global Intelligence Quotient*	92.5 [85.0–98.0]	91.0 [85.0–98.7]	0.999

Legend: experimental group (EG); control group (CG); Wechsler Intelligence Scale for Children-IV (WISC-IV); Evaluation of Dyslexia and Dysorthography (DDE). Variables were expressed as median [first–third quartiles].

**Table 3:** Friedman’s test results and significant differences between experimental and control groups.

	Clinical Assessment	One-Way Repeated-Measures Analysis		Post Hoc Analysis	
		Test Value	*p*-Value	Significant Differences	*p*-Value
**EXPERIMENTAL** **GROUP**	*Word Reading Test (CORRECTNESS)*	18.15	<0.001	T2-T0 T1-T0	<0.001 <0.001
	*No Word Reading Test (CORRECTNESS)*	21.26	<0.001	T2-T0 T1-T0	<0.001 <0.01
	*Text Reading Test (CORRECTNESS)*	17.61	<0.001	T2-T0 T1-T0	<0.05 <0.001
	*Word Writing Test (CORRECTNESS)*	14.33	<0.001	T2-T0 T1-T0	<0.05 <0.001
	*No Word Writing Test (CORRECTNESS)*	21.26	<0.001	T2-T0 T1-T0	<0.001 <0.01
	*Homophone Word Writing Test * *(CORRECTNESS)*	15.27	<0.001	T2-T0 T1-T0	<0.05 <0.001
	*Word Reading Test (SPEED)*	16.97	<0.001	T2-T0 T1-T0	<0.01 <0.001
	*No Word Writing Test (SPEED)*	18.20	<0.001	T2-T0 T1-T0	<0.001 <0.01
	*Text Reading Test (SPEED)*	17.90	<0.001	T2-T0 T1-T0	<0.001 <0.01
**CONTROL** **GROUP**	*Word Reading Test (CORRECTNESS)*	6.22	0.044	T2-T0	<0.05
	*No Word Reading Test (CORRECTNESS)*	4.54	0.103	-	-
	*Text Reading Test (CORRECTNESS)*	14.06	<0.001	T2-T0	<0.001
	*Word Writing Test (CORRECTNESS)*	20.60	<0.001	T2-T0 T1-T0	<0.05 <0.001
	*No Word Writing Test (CORRECTNESS)*	19.63	<0.001	T2-T0 T1-T0	<0.05 <0.001
	*Homophone Word Writing Test * *(CORRECTNESS)*	10.34	0.006	T2-T0	<0.01
	*Word Reading Test (SPEED)*	9.17	0.010	T2-T0	<0.01
	*No Word Writing Test (SPEED)*	10.50	0.005	T2-T0	<0.01
	*Text Reading Test (SPEED)*	5.89	0.052	-	-

## Data Availability

Data are available on the Zenodo Repository DOI: 10.5281/zenodo.12517741.
